# Compound-specific δ^15^N composition of free amino acids in moss as indicators of atmospheric nitrogen sources

**DOI:** 10.1038/s41598-018-32531-x

**Published:** 2018-09-25

**Authors:** Ren-guo Zhu, Hua-Yun Xiao, Zhongyi Zhang, Yuanyuan Lai

**Affiliations:** 1 Jiangxi Province Key Laboratory of the Causes and Control of Atmospheric Pollution, East China University of Technology, Nanchang, 330013 China; 2 College of Water Resources and Environmental Engineering, East China University of Technology, Nanchang, 330013 China; 3 College of Earth Sciences, East China University of Technology, Nanchang, 330013 China

## Abstract

*Haplocladium microphyllum* moss samples were collected in Nanchang, China. Free amino acid (FAA) concentrations and N isotope compositions (δ^15^N_FAA_) in the samples were determined and compared with the bulk N concentrations and δ^15^N_bulk_ values. The aim was to determine whether δ^15^N_FAA_ values in moss (which are very variable) indicate the sources of atmospheric N. The δ^15^N_FAA_ values among individual FAA varied widely (from −19.3‰ to +16.1‰), possibly because of the different sources of N and isotope fractionation in amino acids metabolic pathways. Total ^15^N-enrichment for the individual FAAs was equal to total ^15^N-depletion relative to δ^15^N_bulk_. The concentration-weighted mean δ^15^N value for total FAAs (TFAA) (δ^15^N_TFAA_) was −3.1‰ ± 3.2‰, which was similar to δ^15^N_bulk_ (−4.0‰ ± 2.9‰). We concluded that a N isotope balance occurred during amino acid metabolism and that little isotope disparity occurred between the concentration-weighted TFAA and bulk N. We concluded that δ^15^N_TFAA_ ≈ δ^15^N_bulk_ ≈ δ^15^N_source_. The mean δ^15^N_alanine_ (−4.1‰), δ^15^N_glutamate_ (−4.2‰), and δ^15^N_lysine_ (−4.0‰) were similar to the mean δ^15^N_bulk_, which we attributed to little isotope fractionation occurring during their *in situ* the metabolic pathways. This suggests that δ^15^N_alanine_, δ^15^N_glutamate_, and δ^15^N_lysine_ in moss can be used to indicate the sources of atmospheric N deposition.

## Introduction

Anthropogenic N pollution entering the atmosphere is increasing every year^1^. It is predicted that global anthropogenic N emissions will be 200 Tg y^−1^ by 2050^2^. Atmospheric N deposition can influence soil chemistry^3^, lacustrine and estuarine eutrophication^4^, biological diversity^5^, greenhouse gas balance^6^, and even human health^7^. The levels and sources of atmospheric N deposition urgently need to be assessed.

Due to lack protective cuticles and have large surface areas, moss receives N passively and effectively from atmospheric N deposition^8–11^. Mosses have therefore been used widely to easily and cheaply acquire relatively high spatial resolution data on long-term atmospheric N deposition. Strong relationships between atmospheric N deposition and bulk N concentrations in moss tissues have been found in numerous studies^12–17^.

In field studies, FAA has been found to be more sensitivity to atmospheric N deposition than bulk N in moss^18,19^. Laboratory experiments using ^15^N labelled compounds have indicated that N-containing compounds taken up by moss are immediately assimilated as glutamine (Gln) and then transformed into other FAAs to avoid toxic NH_4_^+^ concentrations accumulating in the cells^20,21^. Strong links between the concentrations of some FAAs and atmospheric N deposition have been found for vascular plants^18,22–27^. However, different types of FAAs have been found to accumulate in different plant species^25,28^. It is still unclear whether FAA concentrations in moss can be used to quantitatively indicate N deposition and which specific FAAs respond most to atmospheric N deposition.

Moss depends on atmospheric N deposition as its main N source and the low isotopic fractionation is accompanied with the uptake of N by moss^13,29,30^. Bulk N isotope compositions (δ^15^N_bulk_) in moss have therefore been used to indicate the dominant sources of N deposition^31–36^. For example, δ^15^N_bulk_ for moss has been found to significantly negatively correlate with the wet deposition NH_4_^+^/NO_3_^−^ ratio^27,37^. However, influences of atmospheric N deposition on N utilization and metabolism of moss were hindered by only analyzing the δ^15^N_bulk_^38,39^. FAAs are important N-containing biomolecules that play central roles in N metabolism in plants and have been shown to be sensitive to atmospheric N pollution^40^. FAA δ^15^N values can be used to help evaluate the responses of N metabolism in plants to environmental N inputs. Bol, *et al*.^41^ found that the histidine (His) and phenylalanine (Phe) δ^15^N values can be used to differentiate functional strategy in relative to acquisition of available N sources. Xu and Xiao^42^ found that the δ^15^N values of some FAAs and total FAAs (TFAA) in needles were depleted when the contribution of traffic was lower. However, Yoneyama and Tanaka^43^ found that a significant isotopic fractionation was connected to the metabolism of FAA in plants. In previous studies differences between δ^15^N_FAA_ values among individual FAA up to 36‰ have been found^38,42^. However, no study explore how δ^15^N_FAA_ pattern reflects atmospheric N source when strong isotope fractionation occurs through FAA metabolism and which specific FAA best reflect the isotope signatures of atmospheric N sources in moss has yet been performed. It is therefore necessary to investigate the relationship between ^15^N abundances in individual FAAs and atmospheric N sources.

In this study, we determined the FAA N concentrations, δ^15^N_FAA_ values, bulk N concentrations and δ^15^N_bulk_ values in moss samples. The FAA and bulk data were compared to determine whether FAAs can be effectively used to assess N deposition. The aims were (1) to assess the relationship between FAA N concentrations and atmospheric N deposition, (2) to determine how to using highly variable δ^15^N_FAA_ values indicate atmospheric N sources, and (3) to determine which specific δ^15^N_FAA_ value best reflects N sources to the atmosphere.

## Results

### Bulk N concentrations and δ^15^N_bulk_

**Figure 1:**
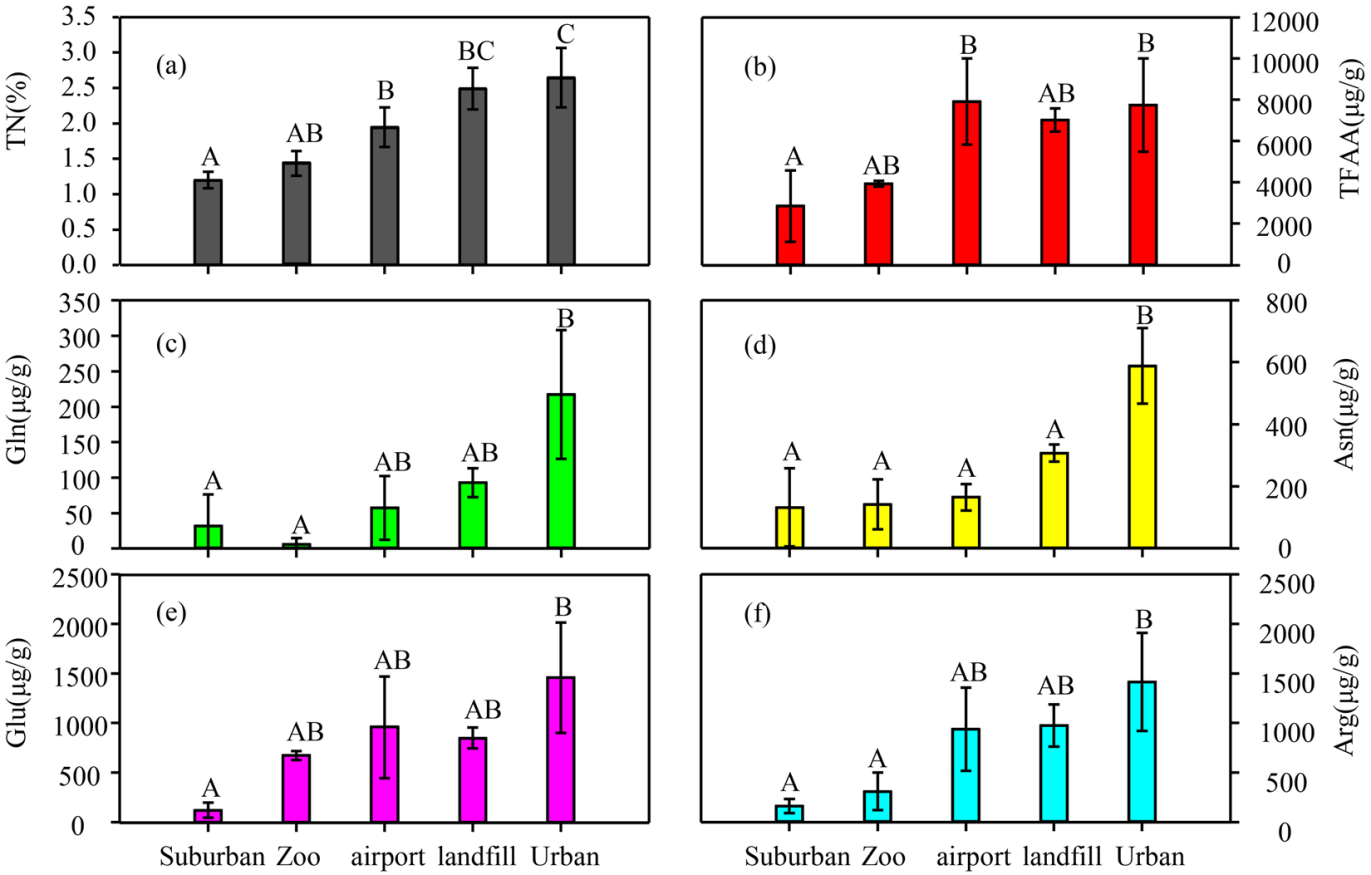
Concentrations of TN, TFAA, Gln, Asn, Glu and Arg in moss from Suburban, Zoo, Airport, Landfill and Urban in Nanchang city: (**a**) TN, (**b**) TFAA, (**c**) Gln, (**d**) Asn, (**e**) Glu and (**f**) Arg. Bars represent mean values ± standard deviations. Significantly different mean values (HSD Tukey’s, p < 0.05) of TN and FAA from different sampling sites are indicated with superscript letters ‘A’ and ‘B’.

The bulk N concentrations in the moss samples were 1.1%–3.0%, and the mean was 1.9% ± 0.6%, as shown in Fig. 1a. The mean bulk N concentrations in moss from the seven sites in Nanchang City were decreased in the order urban centre (2.7% ± 0.4%), landfill (2.5% ± 0.3%), airport (1.9% ± 0.3%), zoo (1.4% ± 0.2%) and suburbs (1.2% ± 0.1%). The mean bulk N concentrations were significant higher in Urban than those in Suburbs (p < 0.05).

**Figure 2:**
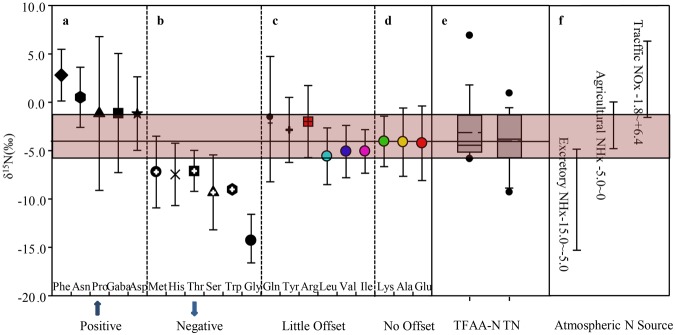
Moss δ^15^N of individual FAAs in NanChang city. The vertical lines represent standard deviations. Moss δ^15^N_TFAA_ and δ^15^N_TN_ were showd in box plot. The box encloses 50% of the data, the whiskers 90% of the data, the solid lines is the median, the dashed line is the mean, solid circles are outliers. The δ^15^N ranges of the potential N sources are also included in the figure. The date of NH_x_ δ^15^N values from excretory wastes is cited from Freyer^59^; Heaton^60^ and Moore^61^. NH_x_ δ^15^N values from agricultural source is referenced from Xiao *et al*.^58^. The δ^15^N value of NO_x_ is cited from Freyer^59^ and Saurer *et al*.^67^.

As shown in Fig. 2, most moss samples had negative mean δ^15^N_bulk_ values. The mean δ^15^N_bulk_ was −4.0‰ ± 2.9‰ and the interquartile range was −5.7‰ to −1.3‰.

### FAA concentrations

The Ala, Arg, Asn, Asp, Gaba, Gln, Glu, Gly, His, Ile, Leu, Lys, Met, Phe, Pro, Ser, Thr, Trp, Tyr, and Val concentrations in the moss samples are shown in Table S1. The concentrations of TFAA (943.9–11100.5 μg g^−1^; Fig. 1b), Gln (not detected to 303.5 μg g^−1^; Fig. 1c), Asn (6.18–750.9 μg g^−1^; Fig. 1d), Glu (49.7–2159.9 μg g^−1^; Fig. 1e), and Arg (114.0–2117.3 μg g^−1^; Fig. 1f) in the samples from the different sites varied in similar ways to the bulk N concentrations (Fig. 1a). The FAA concentrations were significantly higher in the samples from the urban centre than from the suburbs (p < 0.05).

**Figure 3:**
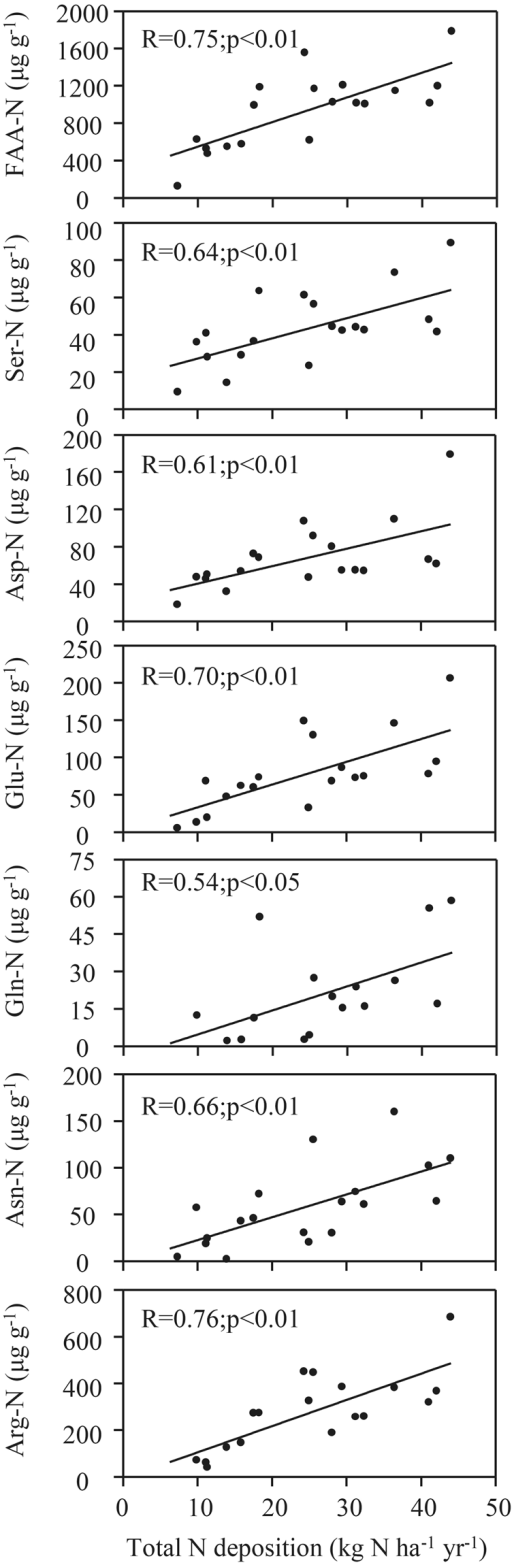
Relationships between concentrations of individual FAA (expressed as N concentrations) in moss and estimated total atmospheric N deposition. C_FAA-N_ calculated by: C_FAA-N_ = C_FAA_∙n∙14. C_FAA_ is the molar concentration of each amino acid; n is the nitrogen atoms contained in each AA; 14 is the relative molecular mass of nitrogen atom. Total atmospheric N deposition (x) at each sampling sites was estimated using the linear correlation equation (y = 0.052x + 0.73, R^2^ = 0.70, P < 0.001; Xiao *et al*.^58^) between atmospheric N deposition (x) values and the corresponding moss TN concentrations (y) from the Yangtze River drainage basin.

The N concentrations of Arg, Asn, Asp, Gln, Glu, Ser, and TFAA strongly positively correlated with atmospheric N deposition (P < 0.05) (Fig. 3). The equations for the relationships between the Arg, Asn, Asp, Gln, Glu, Ser, and TFAA concentrations and atmospheric N deposition are shown in Table S2.

### δ^15^N values for individual FAAs (δ^15^N_FAA_)

**Figure 4:**
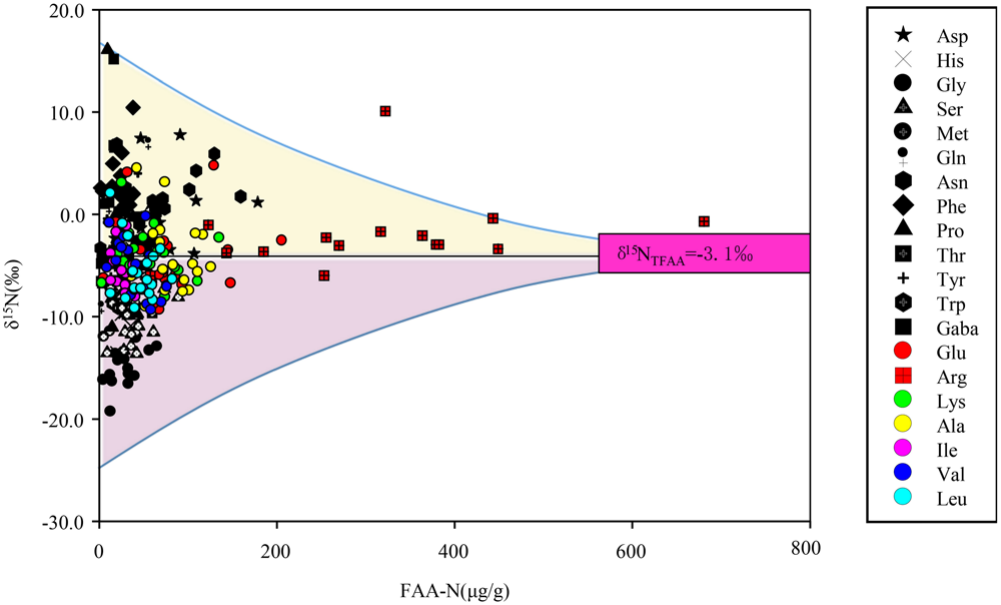
The δ^15^N values of free amino acids (‰) vs. the concentrations of free amino acids (expressed as N concentrations, μg/g) in mosses. δ^15^N_TFAA_ = −3.1‰ is concentration-weighted average nitrogen isotope of free amino acids calculated by Rayleigh equilibrium equation.

The δ^15^N_FAA_ values for the moss samples varied widely, from −19.3‰ to +16.1‰ (Fig. 4). The FAAs were placed in four groups depending on how the δ^15^N_FAA_ compared with the δ^15^N_bulk_ interquartile range. As shown in Fig. 2, the mean δ^15^N values for Ala (−4.1‰), Glu (−4.2‰), and Lys (−4.0‰) (group d) were close to the mean δ^15^N_bulk_ (−4.0‰). The mean δ^15^N values for Arg (−2.0‰), Gln (−1.7‰), Ile (−5.1‰), Leu (−5.6‰), Tyr (−2.9‰), and Val (−5.1‰) (group c) were between the δ^15^N_bulk_ interquartiles (−5.7‰ and −1.3‰). The δ^15^N values for Gly (−14.3‰), His (−7.5‰), Met (−7.2‰), Ser (−9.3‰), Thr (−7.1‰), and Trp (−9.0‰) (group b) were below than the lower δ^15^N_bulk_ quartile. The δ^15^N values for Asn (+0.5‰), Asp (−1.2‰), Gaba (−1.1‰), Phe (+2.8‰), and Pro (−1.2‰) (group a) were higher than the upper δ^15^N_bulk_ quartile.

### Concentration-weighted mean δ^15^N values for the TFAA (δ^15^N_TFAA_)

The FAA δ^15^N values varied widely, there being a 35‰ difference between the highest and lowest, as shown in Fig. 4. The δ^15^N_TFAA_ values were calculated using the isotope mass-balance equation

1 $${{\rm{\delta }}}^{15}{{\rm{N}}}_{{\rm{TFAA}}}=\frac{\sum {{\rm{\delta }}}^{15}{\rm{Ni}}\cdot {\rm{Ci}}\cdot {{\rm{n}}}_{{\rm{i}}}}{\sum {\rm{Ci}}\cdot {{\rm{n}}}_{{\rm{i}}}},$$

where δ^15^N_i_ is the δ^15^N value for FAA i, Ci is the molar concentration of FAA i, and n_i_ is the number of N atoms in FAA i. The mean δ^15^N_TFAA_ was −3.1‰ ± 3.2‰ (Fig. 4) and the interquartile range (−5.2‰ to −1.3‰) was similar to the δ^15^N_bulk_ interquartile range (Fig. 2).

### Fractionation of individual FAA normalized to δ^15^N_bulk_

The method used to calculate positive and negative fractionation of individual FAA normalized to δ^15^N_bulk_ is shown in Fig. S1 The FAAs were divided into three groups depending on the δ^15^N_FAA_ values relative to the mean δ^15^N_bulk_ (−4.0‰). In group δ1, the FAA δ^15^N values were >0‰. In group δ_2_, the FAA δ^15^N values were >−4.0‰ but <0‰. In group δ_3_, the FAA δ^15^N values were <−4.0‰. N isotope fractionation relative to δ^15^N_bulk_ for all three groups was calculated using equation 2.

2 $${{\rm{\Delta }}}^{{\rm{15}}}{\rm{N}}=\frac{\sum ({{\rm{\delta }}}^{{\rm{15}}}{\rm{Ni}}+{\rm{4}}){\rm{Ci}}}{\sum {\rm{Ci}}}$$

**Figure 5:**
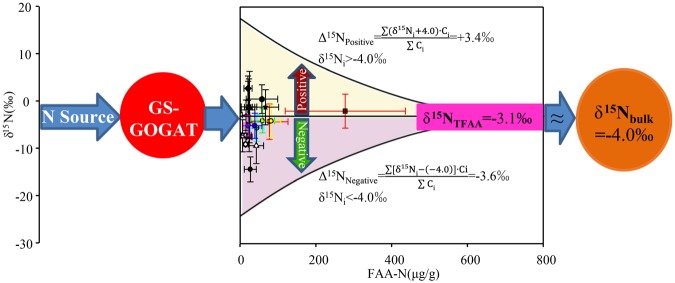
Relationship between δ^15^N_source_, δ^15^N_TFAA_ and δ^15^N_bulk N_. Total ^15^N-enrichment for individual FAA and total ^15^N-depletion *vs*. δ^15^N_bulk_ (−4.0‰) are also showed.

The total positive fractionation relative to δ^15^N_bulk_ (Δ^15^N_positive_ +3.4‰) was equal to the total negative fractionation relative to δ^15^N_bulk_ (Δ^15^N_negative_ −3.6‰) (Fig. 5).

### Spearman correlations between δ^15^N _FAA_, δ^15^N_bulk_, and δ^15^N_TFAA_

Linear regression analyses indicated that the δ^15^N_bulk_ values significantly correlated with the δ^15^N_TFAA_ values and the δ^15^N values for Ala, Gaba, His, Ile, Leu, Lys, and Ser (p < 0.05). δ^15^N_TFAA_ significantly correlated with the δ^15^N values for most of the FAAs (Ala, Arg, Asn, Asp, Gaba, Glu, Gly, His, Ile, Leu, Lys, Pro, Ser, and Val) (p < 0.05). δ^15^N_Glu_ correlated with the δ^15^N values for almost all of the FAAs except for Gaba, Gln, Ile, Met, Phe, Thr, Trp, and Tyr (p < 0.05) (Table S3).

## Discussion

Strong relationships between individual FAA (mainly Arg, Asn, Asp, Glu, Gln, Ser, and TFAA) and atmospheric N deposition have been found in various moss species (Table S2)^19,44–46^. In our study, the concentrations of TFAA and some FAAs also varied spatially in similar ways to the bulk N concentration (Fig. 1) and positively correlated with atmospheric N deposition (Fig. 3). The abilities of FAAs in moss to respond to N inputs are related to the chemical and physiological characteristics of the FAAs. When high N deposition occurs, the Gln, Arg, and Asn concentrations increased because these FAAs have low C:N ratios^40^. Moreover, larger changes in free amino acid concentrations responded to increased atmospheric N additions than total N has been observed in various studies. Baxter, *et al*.^47^ found dramatic transient increases in the concentrations of Arg (by a factor of 19), Asn (by a factor of 4), and Gln (by a factor of 3) in moss exposed to 0.1 mM NH_4_^+^ for 20 d. Huhn and Schulz^18^ found that Arg accumulated much more strongly in Rӧsa (high N concentrations) than in Neuglobsow (low N deposition), the Arg concentration being 150 times higher in moss from Rӧsa than in moss from Neuglobsow. Similarly, in this study, we found that Glu (7-fold), Arg (9-fold), Gln (12-fold) and Asn (4-fold) increased large proportion than total nitrogen (2-fold). The synthesis of N-rich FAAs minimizes carbon use for storing N and avoids toxic concentrations of NH_4_^+^ accumulating in plant tissues^48–51^. Additional metabolic features have been found to be responsible for increases in the concentrations of these FAAs in high nitrogen deposition. For example, Arg is more soluble than other FAAs^19,25,52^, Glu plays a central role in N uptake^18^, Gln increases the photosynthetic capacities of plants^44,53^, and Ser is involved in the photorespiratory N cycle^47^. It is therefore possible that the concentrations, expressed as N concentrations, of some FAAs (Arg, Asn, Asp, Gln, Glu, Ser, and TFAA) in moss could indicate current atmospheric N deposition.

The development of isotopic analysis methods has led to δ^15^N values for amino acids being regarded as important tracers of the sources of and transformation processes affecting N-containing compounds in plant tissues^41,42,54^. However, the δ^15^N values for the 20 FAAs mentioned above were very different, suggesting marked N isotope fractionation occurred during the uptake, translocation, biosynthetic, and metabolic pathways^38,55,56^. Gauthier, *et al*.^38^ found that isotope fractionation between nitrate and Glu gave a δ^15^N value of 15.8‰ and that isotope fractionation associated with Asn synthesis from Asp gave δ^15^N values up to 36‰. In our study, the FAA δ^15^N values for moss covered a wide range, from −19.3‰ to +16.1‰ (Fig. 4). Little or no N isotope fractionation has been assumed to occur during N uptake and translocation in mosses^34^, so the large variations in the FAA δ^15^N values could mainly have been caused by FAA metabolism pathways.

Numerous atmospheric N compounds are directly taken up by moss, and little isotopic fractionation is associated with N assimilation. It has previously been found that δ^15^N_bulk_ values for mosses are good indicators of atmospheric N sources^30,36,57,58^. The mean δ^15^N_bulk_ value for the moss samples from Nanchang City was −4.0‰ ± 2.9‰ (range −9.3‰ to +0.9‰). According to δ^15^N inventories for potential N sources^59–61^, atmospheric N may be deposited in Nanchang mainly as NH_y_ (negative δ^15^N values, group f in Fig. 2) originally emitted in animal excreta (−15.0‰ to −5.0‰)^59–61^ and during agricultural processes (−5‰ to 0‰)^58^. This conclusion was drawn because of the negative δ^15^N values. This result agreed with the results of a previous study of urban, rural, and forested sites in South China^11^. However, most of the δ^15^N_FAA_ values were very different from the δ^15^N_bulk_ values and the δ^15^N_FAA_ range was much wider (35‰) than the δ^15^N_bulk_ range (10‰). It would therefore have been somewhat difficult for the δ^15^N_FAA_ values to indicate the N sources because of isotopic fractionation caused by metabolism, as discussed above.

It has been shown in numerous studies that FAA δ^15^N values are related to fractionation in the FAA metabolic pathways^38,39,43,62–64^. In this study the AA-δ^15^N pattern for free amino acid contrasted to the average value of δ^15^N_TFAA_, to discuss the fractionation with free amino acids metabolic pathways. Compared to the average value of δ^15^N_TFAA_, Gln, Phe, Tyr, Asn and Asp have higher δ^15^N value *vs*. δ^15^N_TFAA_ (Fig. 2). Relative enrichment of ^15^N in Phe has been found to be related to kinetic isotope effects associated with the Phenylalanine ammonia-lyase catalyses Phe deamination, leaving the residual Phe relatively enriched in ^15^N^39,62,65^. Tyr is catalyzed by tyrosine ammonia-lyase to 4-hydroxycinnamate, which associated with marked ^15^N enrichment in Tyr.The δ^15^N value of Pro is positive than the value of δ^15^N_TFAA_, it could be explained by the kinetic isotope effect involved in the catabolism of Pro is greater than that its biosynthesis procedure or the biosynthesis of Pro is an thermodynamic procedure^54^. Relative ^15^N-enrichment in Asp is caused by the transfer of the amino group from Glu to oxaloacetate to form Asp, involving the formation of a protonated Schiff base, favouring ^15^N for Asp production^56^. Styring, *et al*.^54^ attributed ^15^N enrichment in Asn in cereal grains to Asn acting as a transport metabolite. The amino group of Asn is incorporated into other amino acids through transamination with a-keto acids, involving kinetic isotope fractionation discriminating against ^15^N. On the other hand, Gly and Ser have depleted δ^15^N values *vs*. δ^15^N_TFAA_ (Fig. 2). Gly and Ser involve the photorespiratory cycle in the plants. ^15^N-depletion in Gly and Ser was possibly caused by ^15^N-depletion reaction during photorespiration related to Gly and Ser formation, e.g., isotope effect associated with transamination from Glu to produce Gly and discrimination against ^15^N associated with the reaction that converts Gly to Ser^38,63,66^.

Obviously using δ^15^N_FAA_ values to indicate atmospheric N sources could therefore be affected by isotopic fractionation during FAA metabolic reactions in moss, as discussed above. The δ^15^N values for some FAAs may not reliably reflect atmospheric N sources. For example, using ^15^N-enriched FAAs (e.g., Phe, δ^15^N_Phe_ 2.8‰ ± 2.7‰) to identify the main sources of atmospheric N deposition would incorrectly identify the source of N deposition in Nanchang City as being traffic-derived NO_2_ (δ^15^N +1.3‰ to +6.4‰)^67^, whereas using FAAs with more negative δ^15^N values (e.g., Gly, δ^15^N_Gly_ −14.3‰ ± 2.7‰) would indicate the sources being animal excreta (δ^15^N −15.2‰ to −8.9‰) and sewage (δ^15^N −15‰ to −4‰)^59,60^. We attempted to use δ^15^N_TFAA_ as an indicator to solve this. As shown in Fig. 5, the sum of the positive differences between individual δ^15^N_FAA_ values and δ^15^N_bulk_ (Δ^15^N_positive_ +3.4‰) was equal to the sum of the negative differences between individual δ^15^N_FAA_ values and δ^15^N_bulk_ (Δ^15^N_negative_ −3.6‰), implying that the TFAAs were isotopically equilibrated during FAA metabolism in the moss. The mean δ^15^N_TFAA_ (−3.1‰ ± 3.2‰) was close to δ^15^N_bulk_ (−4.0‰ ± 2.9‰), and the δ^15^N_TFAA_ interquartile range (−5.2‰ to −1.3‰) was similar to the δ^15^N_bulk_ interquartile range (−5.7‰ to −1.3‰) (group e in Fig. 2), that is, δ^15^N_TFAA_ ≈ δ^15^N_bulk_ ≈ δ^15^N_Source_. The Pearson correlations indicated that δ^15^N_TFAA_ significantly correlated with δ^15^N_bulk_ (Table S3). We therefore concluded that little isotopic fractionation occurs between TFAA and bulk N, meaning δ^15^N_TFAA_ for moss can be used to indicate atmospheric N sources.

Most δ^15^N_FAA_ values have not been compared with δ^15^N_bulk_ values, so it is not clear which δ^15^N_FAA_ values in moss best indicate N source signatures. Only similar trends in δ^15^N_FAA_ and δ^15^N_sources_ have been reported in previous publications. Chikaraishi, *et al*.^68^ found more ^15^N-depleted FAAs in moss from more industrial areas than in moss from more agricultural areas. Xu and Xiao^42^ found that Ala, Arg, Asp, Glu, His, Ile, Lys, Pro, Ser, and TFAA were more ^15^N-depleted in needles from sites far from highways than in needles from sites near highways, suggesting that atmospheric NHx-N from soil emissions affect δ^15^N_FAA_ values more for needles far from highways than for needles near highways. However, δ^15^N values for most FAAs used as indicators were quite different from δ^15^N values for environmental N sources in a study by Xu and Xiao^42^. They found δ^15^N_Gln_ values < −8‰ for new needles at 800 from the highway. These δ^15^N_Gln_ values may possibly indicating a more ^15^N-depleted N source such as animal excreta (δ^15^N −15‰ to −5‰) rather than NHx-N from soil (δ^15^N −5.8‰ to −3.3‰)^59,60^. If using δ^15^N of specific free amino acid with large fractionation in their metabolism to indicate atmospheric N sources, a misleading conclusion would be obtained. It was unexpected that only a portion of the free amino acid δ^15^N values can hold N source signatures. We compared the δ^15^N_FAA_ to δ^15^N_bulk_ values for the 20 FAAs to identify which δ^15^N values best indicated atmospheric N sources. The mean δ^15^N_Glu_, δ^15^N_Ala_, and δ^15^N_Lys_ values were very similar to the δ^15^N_bulk_ values (Fig. 2). This may have been because no or little isotope fractionation was associated with the metabolic pathways of these FAAs. The main roles of Glu in FAA metabolism in plant tissues are to provide an amino group for the biosynthesis of other amino acids and to receive amino groups from the catabolism of other FAAs, which were confirmed in needles, cereal, pulse, algae and wheat tissues^41,54,69–71^. Our results confirmed this from the N isotope viewpoint in that δ^15^N_Glu_ significantly correlated with the δ^15^N values for most of the FAAs and with δ^15^N_TFAA_ (p < 0.05) (Table S3) and in that δ^15^N_Glu_ (−4.0‰) was similar to δ^15^N_TFAA_ (−3.1‰) (Fig. 2). We also found that the measured δ^15^N_Ala_ value was similar to the measured δ^15^N_Glu_, which would have been because kinetic isotope effects on the biosynthesis of Ala from pyruvate and Glu are weak^39,43,72^. Numerous previously also found that biosynthesizing branched-chain from pyruvate and Glu associated by low kinetic isotope effect^39,72^. Lys displayed no significant offset to the average value of δ^15^N_TFAA_. Gauthier, *et al*.^38^ found that, in plants, Lys acquires N derived from Glu, so δ^15^N_Lys_ will reflect δ^15^N_Glu_. This could help explain why δ^15^N_Lys_ was equal to the mean δ^15^N_bulk_ in our study. The δ^15^N_bulk_ value for moss reliably indicates atmospheric N sources, so we concluded that free Ala, Glu, and Lys (which are little affected by kinetic isotope effects during metabolism) may preserve information on atmospheric N sources.

## Conclusions

The concentrations (expressed as N) of some FAAs (e.g., Arg, Asn, Asp, Gln, Glu, Ser, and TFAA) in moss were positively correlated with total atmospheric N deposition, indicating that the concentrations of those FAAs in moss could indicate atmospheric N deposition with a good degree of sensitivity.

We first used the FAA N isotope compositions to determine whether FAA metabolism in moss could reflect atmospheric N sources. The FAA δ^15^N values for the moss varied widely, probably mainly caused by the FAA metabolic pathways in the moss. However, total FAAs are at isotopic equilibrium during FAA metabolism and that the moss δ^15^N_TFAA_ value could reliably indicate atmospheric N sources. We also found that the δ^15^N values of some FAAs (such as Ala, Glu, and Lys) preserve information on atmospheric N sources as well as δ^15^N_bulk_ preserves this information because little isotope fractionation occurs in the metabolic pathways of these FAAs.

Future work should include an investigation of FAA δ^15^N variability in vascular plants under different N deposition conditions to allow the kinetic isotope effects of N transport in different plant organs to be investigated.

## Materials and Methods

### Sample collection and treatment

**Figure 6:**
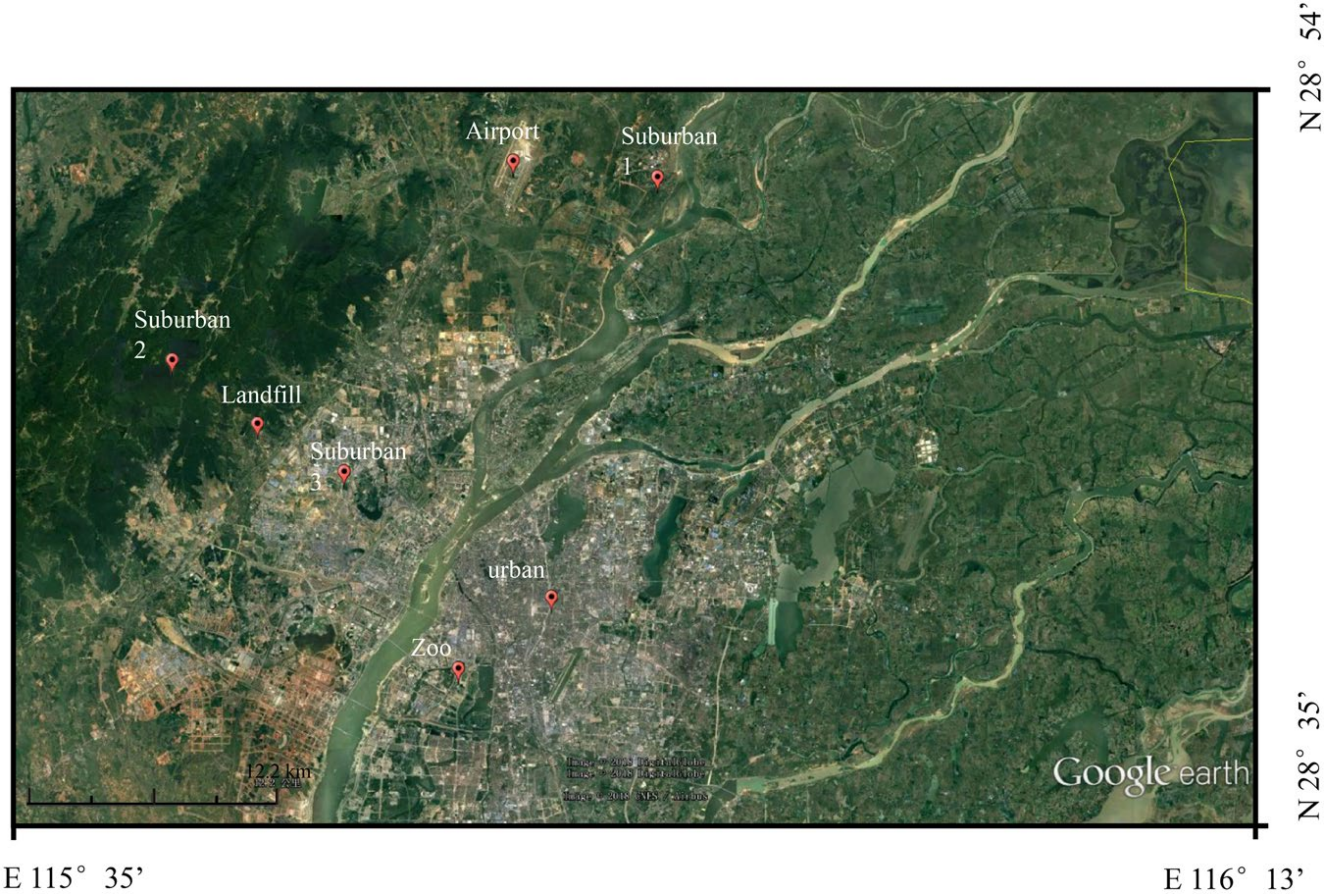
The locations of moss sampling sites in Nanchang city. The locational map was modified from Google Earth 7.1.5.1557 (http://earth.google.com).

*Haplocladium microphyllum* (Hedw.) moss samples were collected from urban, suburban, landfill, and airport sites in Nanchang City (South China) in July 2017. The sampling locations are shown in Fig. 6. Only green, healthy moss was sampled. The sampling sites were chosen based on the results of previous studies^73,74^. Each moss sample was collected from natural rocks in an open field away from overhanging vegetation or tree canopy. Sites were excluded if they could have been affected by point sources of N, such as soil, surface water, or domestic animals. Each sample was collected at least 500 m from any main road and at least 100 m from any other road or a house. Two–four sampling sites were selected in each plot, and 5–10 subsamples were collected at each site, then the subsamples were mixed (to ensure each sample was representative).

Each moss sample was immediately placed in a chilled insulated box. Adsorbed pollutants were removed by gently rinsing each sample with deionized water several times. Half of each washed sample was dried at 80 °C for 1 d, and the other half was freeze-dried. Each dry sample was ground to a fine powder and stored at −80 °C until analysis^68^.

### Bulk N and δ^15^N_bulk_ analyses

The bulk N concentration (expressed in % on a dry weight basis) and δ^15^N_bulk_ were determined simultaneously using a Flash EA 2000 elemental analyser (Thermo Scientific, Bremen, Germany) connected to a Thermo MAT253 plus isotope ratio mass spectrometer (Thermo Scientific, Bremen, Germany). The N concentration analytical precision was better than 0.1%. The δ^15^N method was calibrated by analysing caffeine (IAEA-600, δ^15^N = +1.0‰), ammonium sulfate (USGS25, δ^15^N = −30.4‰), and L-glutamic acid (USGS 41a, δ^15^N = +47.6‰) standards with each set of samples. The δ^15^N analytical precision (standard deviation; n = 3) was better than ±0.05‰. The isotope ratios were expressed in per mil (‰) relative to atmospheric N_2_. Each N concentration and δ^15^N value reported is the mean of at least three measurements.

### FAA extraction, purification, and derivatization

The FAAs were extracted using a method described by Gauthier, *et al*.^38^. Briefly, 0.2–1 g of moss powder was suspended in distilled water, centrifuged for 5 min at 10000 g and 5 °C, then the supernatant was transferred to another centrifuge tube. The sample was extracted again, and the supernatants were mixed and heated to 100 °C for 5 min to precipitate proteins. The extract was then centrifuged for 5 min at 10000 g and 5 °C, then 100 μL of 1 nmol μL^−1^ α-aminobutyric acid was added to act as an internal reference (δ^15^N −8.17‰ ± 0.03‰). The extract was then freeze-dried and resuspended in 1 mL of 0.1 mol L^−1^ HCl. The extract was then passed through a cation exchange column (Dowex 50WX8 H^+^, 200–400 mesh; Sigma-Aldrich, St Louis, MO, USA), and the amino-acid-enriched fraction was stored at −80 °C until analysis.

*tert*-Butyldimethylsilyl (tBDMS)derivatives of the amino acids were prepared following methods described by Molero, *et al*.^55^ and Zhang, *et al*.^75^. Approximately 150 μg anhydrous Na_2_SO_4_, 50 μL pyridine, and 50 μL N-methyl-N-(*tert*-butyldimethylsilyl) trifluoroacetamide were added in sequence to freeze dried amino acids, then the mixture was incubated at 70 °C for 1 h.

### Determining amino acid concentrations and δ^15^N values

Amino acid concentrations and compound-specific structural and δ^15^N values were determined by analysing the *tert*-butyldimethylsilyl derivatives by gas chromatography (GC)/MS/IRMS. The GC/MS/IRMS instrument had a Trace GC instrument (Thermo Fisher Scientific), from which ~10% of the outflow entered a ISQ QD single quadrupole MS instrument (Thermo Fisher Scientific) to allow concentration and structural information to be acquired for each eluting peak. The remaining ~90% of the outflow entered a Thermo GC-isolink, in which the eluted compounds were oxidized and reduced to form CO_2_ and N_2_. The gases then entered a ConFlo IV interface (Thermo Fisher Scientific) and then a Delta V IRMS instrument (Thermo Fisher Scientific) to allow δ^15^N isotope data to be acquired.

The instrument conditions are described below. The injection volume was 0.2–1.0 μL, and splitless mode was used. The autosampler injector temperature was 270 °C. Separation was achieved using a DB-5 column (30 m long, 0.25 mm i.d., 0.25 μm film thickness; Agilent Technologies, Santa Clara, CA, USA). The carrier gas was helium, and the flow rate was 1.0 mL/min. The system was back-flushed with helium for 900 s during each analysis. The GC oven temperature started at 90 °C (held for 1 min), then increased at 12 °C min^−1^ to 150 °C (held for 5 min), increased at 3 °C min^−1^ to 220 °C, then increased at 12 °C min^−1^ to 285 °C (held for 7.5 min). The combustion reactor was held at 1,000 °C.

The linearity of the GC/MS method was assessed by evaporating, derivatizing, and analysing a series of standards containing 20 amino acids at concentrations of 0.04–1 mM. Each standard contained alanine (Ala), γ-aminobutyric acid (Gaba), arginine (Arg), asparagine (Asn), aspartate (Asp), glutamine (Gln), glutamate (Glu), glycine (Gly), histidine (His), isoleucine (Ile), leucine (Leu), lysine (Lys), methionine (Met), phenylalanine (Phe), prolineb (Pro), serine (Ser), threonine (Thr), tryptophan (Trp), tyrosine (Tyr), and valine (Val). The concentration of each amino acid was determined from the GC-MS signal using the relevant calibration curve produced from the standard amino acid mix data and corrected for the α-aminobutyric acid recovery. The R^2^ values for the calibration curves were 0.9909–0.9969, indicating that the method was accurate.

A derivatized mixture of 20 amino acid standards and several single amino acid standards (Ala Gly3, Gly4, Phe, USGS40, USGS41a, and Val) with known δ^15^N values (−26.35 to +47.55‰) was prepared to allow instrumental performance to be monitored and drift to be corrected. The amino acids were successfully converted into TBDMS derivatives and could be completely resolved by GC-C-IRMS (Fig. S2). The results are shown in Table S4. The α-aminobutyric acid (internal standard) δ^15^N value for each sample was used to confirm that the isotope measurements were reproducible. The 20 amino acid standard mixture was analysed after every three samples during a GC/MS/IRMS run to assess the isotope measurement reproducibility and normalize the δ^15^N values of the amino acids in the samples^42^. The amount of sample analysed by GC/MS/IRMS needed to be considered. Standard mixture containing the 20 amino acids each at an equivalent of 0.8 nmol (equivalent to FAA concentration of 9–20 μg g^−1^ in moss) was analysed to allow the δ^15^N values of low concentrations of amino acids to be determined. The FAA concentrations expressed as N concentrations in our samples were higher than these concentrations. The δ^15^N measurement precisions (n = 9) for the derivatized amino acid standard mixtures were 0.5‰–1.4‰ (Table S4). The δ^15^N values for the underivatized amino acids measured by elemental analysis/IRMS correlated with the δ^15^N values for the derivatized amino acids measured by GC/MS/IRMS (R^2^ = 0.997, P < 0.001). The differences between the empirically corrected δ^15^N values measured by elemental analysis/IRMS and GC/MS/IRMS were 0.1‰–1.3‰ (Table S4). Each value reported here is the mean of at least three δ^15^N determinations.

### Statistical analysis

Statistical analyses were performed using SPSS 16.0 software (IBM, Armonk, NY, USA). The statistical significances of differences in the FAA contents of samples from different sites were tested using the one-way analysis of variance method and Tukey-HSD tests, and differences were considered significant at P < 0.05. Correlations between δ^15^N_FAA_, δ^15^N_TFAA_, and δ^15^N_bulk_ were assessed using Pearson correlation coefficients (r). Linear regressions were used to identify correlations between the FAA concentrations and estimated atmospheric N deposition. Most graphs were drawn using SigmaPlot 10.0 software (Systat Software, San Jose, CA, USA).

## Electronic supplementary material

Supplementary information

## Data Availability

All data generated or analyzed during this study are included in this published article and its Supplementary Information file.
