# Supplementary material for: Compound-specific δ15N composition of free amino acids in moss as indicators of atmospheric nitrogen sources
Source: Sci Rep. 2018 Sep 25;8:14347. doi: 10.1038/s41598-018-32531-x (PMC6156404; doi:10.1038/s41598-018-32531-x)
Supplement: Supplementary file 1 — Supplementary information [file 41598_2018_32531_MOESM1_ESM.docx]

**SUPPORTING INFORMATION**

**Compound-specific δ^15^N composition of free amino acids in moss as indicators of atmospheric nitrogen sources**

Ren-guo Zhu^1,2^, Hua-Yun Xiao^1,2,*^, Zhongyi Zhang^1,2^, Yuanyuan Lai^1,3^

1 Jiangxi Province key Laboratory of the Causes and Control of Atmospheric pollution, East China University of Technology, Nanchang 330013, China

2 College of Water Resources and Environmental Engineering, East China University of Technology, Nanchang 330013, China

3 College of Earth Sciences, East China University of Technology, Nanchang 330013, China

Correspondence to: Hua-Yun Xiao ([xiaohuayun@ecit.cn](mailto:xiaohuayun@ecit.cn))

# Supplementary Information Contents:

In total 7 pages including:

Tables: 4 Tables (pages 2−6)

Figures: 2 figures (pages 7-8).

References: References for the supplementary information (pages 9)

**Table S1.** The concentrations and δ^15^N values of Ala, Gly, Val, Leu, Ile, Gaba, Pro, Met, Ser, Thr, Phe, Asp, Glu, Asn, Lys, Gln, Arg, His, Tyr and Trp in moss tissues.

| Name | Abbreviation | Concentrations (μg/g) | | δ^15^N(‰) | |
| --- | --- | --- | --- | --- | --- |
|  |  | Mean | Std. Deviation | Mean | Std. Deviation |
| Alanine | Ala | 523.3 | 161.4 | -4.1 | 3.5 |
| Glycine | Gly | 146.6 | 88.5 | -14.3 | 2.7 |
| Valine | Val | 328.4 | 161.2 | -5.1 | 2.7 |
| Leucine | Leu | 421.1 | 172.0 | -5.6 | 2.9 |
| Isoleucine | Ile | 201.5 | 106.6 | -5.1 | 2.3 |
| γ-aminobutyric acid | Gaba | 175.1 | 154.9 | -1.1 | 6.2 |
| Proline | Pro | 207.0 | 113.5 | -1.2 | 7.9 |
| Methionine | Met | 65.6 | 46.6 | -7.2 | 3.7 |
| Serine | Ser | 323.0 | 146.8 | -9.3 | 3.9 |
| Threonine | Thr | 133.0 | 119.1 | -7.1 | 2.1 |
| Phenylalanine | Phe | 235.7 | 136.2 | 2.8 | 2.7 |
| Aspartate | Asp | 631.8 | 331.7 | -1.2 | 3.8 |
| Glutamate | Glu | 815.4 | 530.0 | -4.2 | 3.9 |
| Asparagine | Asn | 273.0 | 201.2 | 0.5 | 3.1 |
| Lysine | Lys | 309.7 | 170.9 | -4.0 | 2.6 |
| Glutamine | Gln | 105.1 | 97.7 | -1.7 | 6.5 |
| Arginine | Arg | 863.6 | 508.1 | -2.0 | 3.7 |
| Histidine | His | 90.1 | 28.4 | -7.5 | 3.2 |
| Tyrosine | Tyr | 207.3 | 131.9 | -2.9 | 3.4 |
| Tryptophan | Trp | 106.4 | 56.1 | -9.0 | 0.2 |

T**able S2.** The relationship between N concentrations of FAAs and total N deposition in this and previous studies.

| Species | Amino acids | Relationship | ANOVA,P | Equation | Reference |
| --- | --- | --- | --- | --- | --- |
| *Haplocladium microphyllum* (Hedw.) | **Asn** | 0.43 | 0.0022 | y=-1.86+2.44x | this study |
| *Haplocladium microphyllum* (Hedw.) | **Gln** | 0.34 | 0.0133 | y=-5.01+0.97x | this study |
| *Haplocladium microphyllum* (Hedw.) | **Arg** | 0.58 | 0.0003 | y=-8.27+11.23x | this study |
| *Haplocladium microphyllum* (Hedw.)  *Haplocladium microphyllum* (Hedw.)  *Haplocladium microphyllum* (Hedw.) | **Glu**  **Asp**  **Ser** | 0.48  0.37  0.40 | 0.001  <0.01  <0.01 | y=2.98+3.04x  y=21.55+1.87x  y=16.58+1.08x | this study  this study  this study |
| *Haplocladium microphyllum* (Hedw.) | **TFAA** | 0.48 | 0.0011 | y=283.4+26.39x | this study |
|  |  |  |  |  |  |
| *Dicranum majus* Sm. | **Asn** |  | 0.01 |  | Nordin *et al.*, 1998 |
| *Dicranum majus* Sm. | **Gln** |  | 0.009 |  | Nordin *et al.*, 1998 |
| *Dicranum majus* Sm. | **Arg** |  | 0.008 |  | Nordin *et al.*, 1998 |
| *Dicranum majus* Sm. | **TFAA** |  | 0.01 |  | Nordin *et al.*, 1998 |
|  |  |  |  |  |  |
| *Pleurozium schreberi*(Brid.) Mitt. | **Asn** |  | 0.009 |  | Nordin *et al.*, 1998 |
| *Pleurozium schreberi*(Brid.) Mitt. | **Gln** |  | 0.026 |  | Nordin *et al.*, 1998 |
| *Pleurozium schreberi*(Brid.) Mitt. | **Arg** |  | 0.001 |  | Nordin *et al.*, 1998 |
| *Pleurozium schreberi*(Brid.) Mitt. | **TFAA** |  | 0.001 |  | Nordin *et al.*, 1998 |
| *Sphagnum*  *Sphagnum*  *Sphagnum*  *Sphagnum*  *Sphagnum*  *Sphagnum*  *Sphagnum* | **Arg**  **Asn**  **Gln**  **Asp**  **Glu**  **Ser**  **TFAA** |  | <0.001  <0.001  <0.001  <0.05  <0.01  <0.001  <0.001 |  | Nordin *et al.*, 2000  Nordin *et al.*, 2000  Nordin *et al.*, 2000  Nordin *et al.*, 2000  Nordin *et al.*, 2000  Nordin *et al.*, 2000  Nordin *et al.*, 2000 |
| *Sphagnum magellanicum* | **Asn** | 0.6 | 0.002 |  | Limpens and Berendse, 2003 |
| *Sphagnum magellanicum* | **Gln** | 0.44 | 0.013 |  | Limpens and Berendse, 2003 |
| *Sphagnum magellanicum* | **Arg** | 0.74 | 0.001 |  | Limpens and Berendse, 2003 |
| *Sphagnum fallax* | **Arg** |  | <0.05 |  | Tomssen & Roelofs, 2003 |
| *Sphagnum fallax* | **Asn** |  | <0.05 |  | Tomssen & Roelofs, 2003 |
| *Sphagnum fallax*  *Sphagnum fallax*  *Betula pubescens*  *Betula pubescens*  *Molinia caerulea*  *Molinia caerulea*  *Molinia caerulea* | **Gln**  **Glu**  **Arg**  **Asp**  **Gln**  **Asp**  **Ser** |  | <0.05  <0.05  <0.05  <0.05  <0.05  <0.05  <0.05 |  | Tomssen & Roelofs, 2003  Tomssen & Roelofs, 2003  Tomssen & Roelofs, 2003  Tomssen & Roelofs, 2003  Tomssen & Roelofs, 2003  Tomssen & Roelofs, 2003  Tomssen & Roelofs, 2003 |

**Table S3.** Matrix of correlation between the δ^15^N values of bulk N, TFAA and 20 specific free amino acids. * Correlation is significant at the 0.05 level (2-tailed). ** Correlation is significant at the 0.01 level (2-tailed).

|  | TN | TFAA | Ala | Gly | Val | Leu | Ile | Gaba | Pro | Met | Ser | Thr | Phe | Asp | Glu | Asn | Lys | Gln | Arg | His | Tyr | Trp |
| --- | --- | --- | --- | --- | --- | --- | --- | --- | --- | --- | --- | --- | --- | --- | --- | --- | --- | --- | --- | --- | --- | --- |
| TN | 1 | * | * |  |  | ** | * | * |  |  | ** |  |  |  |  |  | ** |  |  | ** |  |  |
| TFAA | * | 1 | ** | * | ** | ** | * | * | * |  | ** |  |  | ** | ** | ** | ** |  | ** | ** |  |  |
| Ala | * | ** | 1 | ** | ** | ** |  | * |  |  | ** |  |  | ** | ** | ** | ** |  | ** | ** |  |  |
| Gly |  | * | ** | 1 |  | ** |  |  |  |  |  |  |  | * | * |  |  |  |  | * |  |  |
| Val |  | ** | ** |  | 1 | ** |  |  |  |  | ** |  | * | * | ** |  | ** |  | * | ** |  |  |
| Leu | ** | ** | ** | ** | ** | 1 | * |  |  |  | ** |  |  | ** | ** | * | ** |  | ** | ** |  |  |
| Ile | * | * |  |  |  | * | 1 | * |  |  |  | * | * | ** |  |  |  |  |  | ** |  |  |
| Gaba | * | * | * |  |  |  | * | 1 |  |  |  |  |  | * |  |  | ** |  |  | ** |  | ** |
| Pro |  | * |  |  |  |  |  |  | 1 |  |  |  |  | * | * |  |  |  | * |  |  |  |
| Met |  |  |  |  |  |  |  |  |  | 1 |  |  |  |  |  |  |  | * |  |  | * |  |
| Ser | ** | ** | ** |  | ** | ** |  |  |  |  | 1 |  |  | ** | ** | ** | ** |  | * | ** |  |  |
| Thr |  |  |  |  |  |  | * |  |  |  |  | 1 |  |  |  |  | * |  |  |  |  |  |
| Phe |  |  |  |  | * |  | * |  |  |  |  |  | 1 |  |  |  |  |  |  |  |  |  |
| Asp |  | ** | ** | * | * | ** | ** | * | * |  | ** |  |  | 1 | ** | ** | ** |  | ** | ** |  |  |
| Glu |  | ** | ** | * | ** | ** |  |  | * |  | ** |  |  | ** | 1 | ** | ** |  | ** | * |  |  |
| Asn |  | ** | ** |  |  | * |  |  |  |  | ** |  |  | ** | ** | 1 | * |  | ** |  |  | * |
| Lys | ** | ** | ** |  | ** | ** |  | ** |  |  | ** | * |  | ** | ** | * | 1 |  | ** | ** |  |  |
| Gln |  |  |  |  |  |  |  |  |  | * |  |  |  |  |  |  |  | 1 |  |  |  |  |
| Arg |  | ** | ** |  | * | ** |  |  | * |  | * |  |  | ** | ** | ** | ** |  | 1 | * |  |  |
| His | ** | ** | ** | * | ** | ** | ** | ** |  |  | ** |  |  | ** | * |  | ** |  | * | 1 |  | ** |
| Tyr |  |  |  |  |  |  |  |  |  | * |  |  |  |  |  |  |  |  |  |  | 1 |  |
| Trp |  |  |  |  |  |  |  | ** |  |  |  |  |  |  |  | * |  |  |  | ** |  | 1 |

**Table S4.** Comparison of δ^15^N values for high purity amino acid standard and international amino acid standard using the EA/IRMS and GC/C/IRMS methods. The measurement precision and accuracy is for samples with 0.8 nmol.

| Amino acid | Abbreviation | δ^15^N_nat_^a^  (‰) | SD^b^ | δ^15^N^c^  (‰) | SD^d^ | Δ^e^ |
| --- | --- | --- | --- | --- | --- | --- |
| High purity amino acid standard | | | | | | |
| Alanine | Ala | -1.20 | 0.21 | -0.5 | 0.5 | -0.7 |
| Glycine | Gly | 1.15 | 0.09 | 1.4 | 0.8 | -0.3 |
| Valine | Val | -3.45 | 0.06 | -3.0 | 0.6 | -0.4 |
| Leucine | Leu | 6.54 | 0.05 | 6.2 | 1.1 | 0.4 |
| Isoleucine | Ile | 1.29 | 0.14 | 1.6 | 1.0 | -0.3 |
| γ-aminobutyric acid | Gaba | -7.11 | 0.22 | -5.8 | 0.9 | -1.3 |
| Proline | Pro | -3.20 | 0.21 | -4.0 | 1.4 | 0.8 |
| Methionine | Met | -2.48 | 0.19 | -1.9 | 0.5 | -0.6 |
| Serine | Ser | 1.34 | 0.09 | 1.3 | 0.9 | 0.1 |
| Threonine | Thr | -5.11 | 0.06 | -5.7 | 1.3 | 0.6 |
| Phenylalanine | Phe | 16.26 | 0.12 | 16.0 | 0.7 | 0.2 |
| Asparatic acid | Asp | -2.04 | 0.07 | -1.6 | 0.7 | -0.5 |
| Glutamic acid | Glu | -5.15 | 0.14 | -6.3 | 1.3 | 1.2 |
| Asparagine | Asn | 1.46 | 0.09 | 2.3 | 1.1 | -0.8 |
| Lysine | Lys | 0.02 | 0.18 | -0.4 | 0.7 | 0.5 |
| Glutamine | Gln | -2.76 | 0.16 | -2.3 | 1.0 | -0.5 |
| Arginine | Arg | -5.88 | 0.14 | -4.6 | 0.6 | -1.3 |
| Histidine | His | -7.21 | 0.10 | -7.8 | 0.7 | 0.6 |
| Tyrosine | Tyr | 5.06 | 0.20 | 3.8 | 1.2 | 1.2 |
| Tryptophan | Trp | -3.33 | 0.12 | -3.8 | 0.7 | 0.5 |
| Reference material of known isotope composition | | | | | | |
| Valine | Val* | 30.08 | 0.02 | 31.1 | 0.6 | -1.0 |
| Phenylalanine | Phe* | 1.70 | 0.06 | 2.1 | 0.5 | -0.4 |
| Glycine | Gly3* | 40.83 | 0.02 | 41.9 | 0.1 | -1.1 |
| Glycine | Gly4* | -26.35 | 0.02 | -26.9 | 0.5 | 0.5 |
| Alanine | Ala* | 43.25 | 0.07 | 42.8 | 0.4 | 0.4 |
| Glutamic acid | USGS40# | -4.52 |  | -5.3 | 0.2 | 0.8 |
| Glutamic acid | USGS41# | 47.55 |  | 46.5 | 0.3 | 1.1 |
| ^a^Measured by EA/IRMS (n=10). | | | | | | |
| ^b^Measured by GC/C/IRMS (n=9). | | | | | | |
| ^c^The precision of EA-IRMS. | | | | | | |
| ^d^The precision of GC-C-IRMS. | | | | | | |
| ^e^: Difference in δ^15^N values determined by EA/IRMS and GC/C/IRMS. | | | | | | |
| ^*^ commercially available from Indiana University | | | | | | |
| ^#^ Purchased from the International Atomic and Energy Agency | | | | | | |

**Figure S1.** The method calculating fractionation of individual δ^15^N_FAA_ relative to δ^15^N_bulk_ in moss. 20 FAAs were divided to 3 groups by comparing their δ^15^N values to the average values of δ^15^N_bulk_ (-4.0‰). The δ^15^N values of the first group were higher than 0‰. The fractionation of individual FAA against TFAA in first group demonstrated as δ_1_. δ_1_=4.0+δ^15^N_i_. The second group was those which δ^15^N values of individual FAA were higher than -4.0‰ and below than 0‰ (δ_2_). δ_2_=4.0+δ^15^N_i_. The third group was those which δ^15^N values of individual FAA were below than -4.0‰ (δ_3_). δ_3_=δ^15^N_i_-(-4.0). Δ^15^N_positive_ is the sum of ^15^N-enrichment of individual FAA against to the δ^15^N_bulk_, including δ^15^N values of individual FAA higher than -4.0‰ (δ_1_ and δ_2_); Δ^15^N_negative_ is the sum of ^15^N-depletion of individual FAA relative to the δ^15^N_bulk_, including δ^15^N values of individual FAA lower than -4.0‰ (δ_3_). Δ^15^N_positive_ and Δ^15^N_negative_ can be calculated by the same equation: $\text{∆}\text{15}\text{N=}\frac{\sum\left( \text{δ}\text{15}\text{N}\text{i}\text{+}\text{4.0} \right)\text{C}\text{i}}{\sum\text{C}\text{i}}$.

**Figure S2.** GC-C-IRMS chromatogram for the analysis of amino acid standard mixture tBDMSi derivatives. Peaks: 1 Ala; 2 Gly; 3 I.S. (Αaba); 4 Val; 5 Leu; 6 Ile; 7 Gaba; 8 Pro; 9 Met; 10 Ser; 11 Thr; 12 Phe; 13 Asp; 14 Glu; 15 Asn;16 Lys; 17 Gln; 18 Arg; 19 His; 20 Tyr; 21 Trp (2TMS); 22 Trp (3TMS).

# References

Limpens J. & Berendse F. (2003) Growth reduction of Sphagnum magellanicum subjected to high nitrogen deposition: the role of amino acid nitrogen concentration. *Oecologia*, **135**, 339-345.

Nordin A. & Gunnarsson U. (2000) Amino acid accumulation and growth of Sphagnum under different levels of N deposition. E*coscience*, **7**, 474-480.

Nordin A., Nasholm T. & Ericson L. (1998) Effects of Simulated N Deposition on Understorey Vegetation of a Boreal Coniferous Forest. *Functional Ecology*, **12**, 691-699.

Tomassen H.B.M. & Roelofs J.G.M. (2003) Stimulated Growth of Betula pubescens and Molinia caerulea on Ombrotrophic Bogs: Role of High Levels of Atmospheric Nitrogen Deposition. *Journal of Ecology*, **91**, 357-370.
